# Comparative investigation of binary and ternary hybrid nanocomposites synthesized from a nanoporous organic matrix

**DOI:** 10.1039/d5ra05251c

**Published:** 2025-09-09

**Authors:** N. Ben Mansour, M. Hjiri, N. Mustapha, Fatemah M. Barakat, Talal F. Qahtan, L. El Mir

**Affiliations:** a Laboratory of Physics of Materials and Nanomaterials Applied at Environment (LaPhyMNE), Gabes University, Faculty of Sciences in Gabes Gabes Tunisia nabil.benmansour@ipeigb.rnu.tn Benmansour.nabil@yahoo.fr +0021697901939; b Departement of Physics, College of Sciences, Imam Mohammad Ibn Saud Islamic University (IMSIU) Riyadh 11623 Saudi Arabia; c Physics and Astronomy Department, Faculty of Science, King Saud University Riyadh Saudi Arabia; d Physics Department, College of Science and Humanities in Al-Kharj, Prince Sattam Bin Abdulaziz University Al-Kharj 11942 Saudi Arabia

## Abstract

A nanoporous organic matrix (NPOM) based on pyrogallol-formaldehyde (PF) resin, a binary hybrid nanocomposite (BHyNC) obtained by incorporation of nickel oxide (NiO) nanoparticles into the PF network, and a ternary hybrid nanocomposite (THyNC), resulting from the co-integration of NiO and silica (SiO_2_) nanoparticles within the same network structure were synthesized *via* the sol–gel process. The resulting materials were subsequently subjected to thermal treatment at 650 °C for two hours in a tubular furnace under an inert atmosphere. The XRD diffractograms display broad diffraction peaks characteristic of silica and carbon phases, along with three distinct nickel peaks observed in both BHyNC and THyNC. XPS analysis identifies five prominent peaks in the different samples corresponding to Si 2p, Si 2s, C 1s, O 1s and Ni 2p. SEM micrographs reveal a high density of particles covering the OM, particularly in the THyNC. TEM images reveal a highly porous structure in the organic matrix, a uniform dispersion of nickel nanoparticles in the BHyNC and significant nanoparticle agglomeration in the THyNC. The Raman spectra confirm the presence of a disordered graphitic structure in the various materials, as demonstrated by the D to G band intensity ratio, which ranges between 0.7 and 1. The conductance of the materials is influenced by the pore volume, showing a decrease as the pore volume increases. Electrochemical measurements demonstrate that the sensitivity of the non-enzymatic glucose sensor increases as the specific surface area decreases. According to the properties of the obtained materials, THyNC exhibited the most promising electrochemical performance as a non-enzymatic glucose sensor, whereas NPOM and BHyNC showed favorable properties for low temperature electronic applications.

## Abstract 2

THyNC showed superior performance as a non-enzymatic glucose sensor, while NPOM and BHyNC demonstrated potential for low-temperature electronic applications.

## Graphical abstract

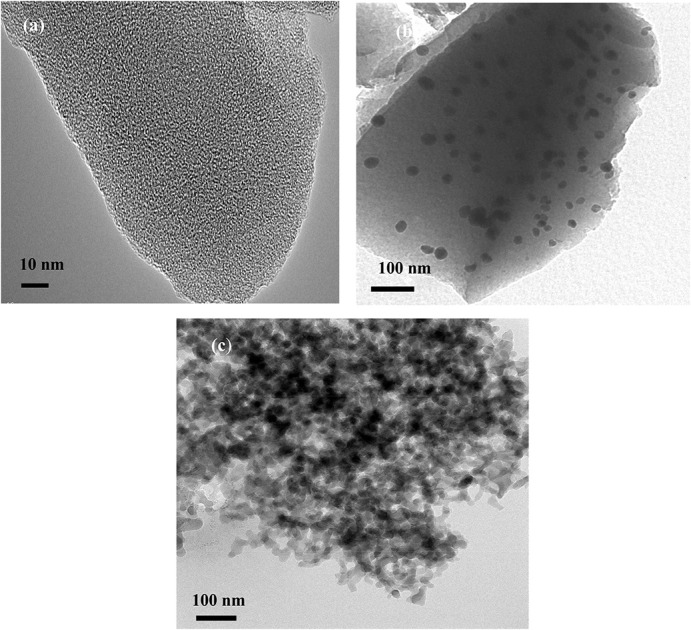

## 1. Introduction

Nanoporous materials are increasingly recognized as highly versatile, capturing the attention of researchers thanks to their unique structural and functional properties. Typically defined by pore diameters of less than 100 nanometers, these materials offer an exceptional combination of large specific surface area, low density and excellent resistance to high temperatures. Such features render nanoporous materials indispensable across numerous technological fields, including gas separation, chemical sensing and energy storage.^1–6^ Their outstanding performance is largely linked to their capacity to facilitate interactions with molecules and ions in confined spaces. Importantly, these interactions can be specifically tailored through accessible and cost-effective synthesis methods.

Nanoporous materials are generally categorized into three main types based on pore size: microporous (pore diameter <2 nm), mesoporous (2–50 nm), and macroporous (>50 nm). Among these, materials based on an organic matrix, such as hybrid nanocomposites have gained increasing attention in recent years.^7–10^ These hybrid materials offer several advantages, including broad synthetic flexibility and reduced manufacturing costs. Their performance in applications like gas storage and separation is largely determined by their specific surface area and pore volume, which can be comparable to those of conventional inorganic microporous frameworks. Over the past two years, significant progress has been achieved in the design of hybrid nanocomposites, highlighting their improved electrochemical performance and stability.^11–13^ Hybrid nanocomposites can be categorized into binary systems, comprising a single class of inorganic nanoparticles dispersed within an organic matrix and ternary systems, formed by the simultaneous incorporation of two distinct inorganic nanoparticle species into the organic framework. Very recent studies (2023–2025) have shown that incorporating two distinct inorganic components into an organic matrix provides synergistic effects that substantially enhance electrochemical performance compared to binary or single-component systems.^14–16^ This ternary architecture facilitates the simultaneous optimization of critical parameters such as electrical conductivity, structural integrity and charge storage capacity.^17–19^ The latest developments in ternary nanocomposites (2023–2025) combining metal oxides, conductive polymers and carbon based materials demonstrate remarkable improvements in cycling stability and energy storage capacity compared to earlier designs.^20–22^ Such multifunctional materials are particularly promising for applications requiring high energy density, including electric vehicles, grid-level renewable energy storage and portable electronic devices. Owing to their superior electrochemical kinetics and augmented multifunctional properties, ternary nanocomposites are positioned as prime candidates for integration into advanced energy storage systems.^23,24^

Nickel based hybrid nanocomposites have recently attracted considerable interest due to their remarkable electrocatalytic activity in glucose oxidation, as well as their ability to enhance the electrical conductivity of organic matrices.^25–27^ Indeed, the electrocatalytic performance of nickel based sensors is primarily attributed to the Ni^2+^/Ni^3+^ redox couple, derived from the Ni(OH)_2_/NiOOH system that forms on the surface of the working electrode under alkaline conditions.^28–30^ Moreover, nickel serves as an efficient catalyst for the synthesis of graphitic carbon structures, which contribute to improved electrical conductivity of the hybrid material.^31–34^ Silica nanoparticles have been effectively used to immobilize various biomolecules due to their large specific surface area and strong surface immobilization capabilities.^35–37^ However, when present alone within the composite, they are not ideal candidates for the fabrication of glucose sensors. In this context, the interactions between glucose molecule and the nanoparticle surface are primarily governed by adsorption and electrostatic forces, which limit the active surface for sensing. Conversely, incorporating silica into a composite enhances its stability and provides a robust framework for the uniform dispersion of nickel nanoparticles, thereby improving glucose sensing performance.^38,39^ Moreover, it fills the nanopores and provides a stable support for the uniform dispersion of nickel nanoparticles on the surface. This structural configuration markedly enhances the glucose sensing performance of the ternary hybrid nanocomposite sensor by increasing its active surface and reduce its electrical conductivity.

In this study, we explore the effect of incorporating NiO nanoparticles and the co-incorporation of NiO/SiO_2_ nanoparticles on the properties of a nanoporous organic matrix (NPOM) derived from pyrogallol-formaldehyde resin. The binary hybrid nanocomposite (BHyNC) refers to the system containing only NiO, while the ternary hybrid nanocomposite (THyNC) involves the simultaneous incorporation of both NiO and SiO_2_. The objective is to assess how these inorganic additives influence the structural, morphological, vibrational, electrical and electrochemical properties of the NPOM. Particular attention is given to the impact of these modifications on electrical conductivity and glucose sensing performance. These functional properties are analyzed in relation to key textural parameters, including specific surface area and pore volume to better understand the texture property relationships governing electrical conduction and glucose sensor performance.

## 2. Experimental

### 2.1. Synthesis protocol

The synthesis of the materials was conducted in three stages corresponding to the nanoporous organic matrix (NPOM), the binary hybrid nanocomposite (BHyNC) and the ternary hybrid nanocomposite (THyNC). The preparation of NPOM was carried out in three main steps. First, organic xerogels were synthesized by mixing formaldehyde (F) with pyrogallol (P) dissolved in water (W), using picric acid as a catalyst. The solution was magnetically stirred for 30 minutes. The stoichiometric molar ratios of P/F and P/W were fixed at 1/3 and 1/6, respectively. In the second step, the resulting wet gel was aged and dried in a humid atmosphere at 50 °C for two weeks. Finally, to obtain the NPOM xerogel, the wet gel was transferred into an incubator and further dried at 150 °C with a heating rate of 10 °C per day, after which the final temperature was maintained for two additional days.

NiO nanoparticles were synthesized *via* a sol–gel method with supercritical ethanol drying following the procedure of Ben Mansour *et al.*^40–44^ Nickel(ii) chloride (NiCl_2_·6H_2_O) was dissolved in methanol and stirred for 15 minutes at room temperature. The resulting solution was then transferred into an autoclave and dried under the supercritical conditions of ethanol (*T*_c_ = 250 °C, *P*_c_ = 7 MPa). In the second step, the obtained aerogel was annealed in air at 500 °C for two hours in a muffle furnace. These NiO nanoparticles were incorporated into the pyrogallol-formaldehyde matrix at a 5% mass ratio, followed by conventional drying to yield the BHyNC. Silica nanoparticles were prepared similarly *via* sol–gel with supercritical drying using tetraethoxysilane (TEOS) and hydrofluoric acid as catalyst, dried under supercritical conditions of ethanol to form an aerogel and calcined at 500 °C two hours in a muffle furnace. This furnace is equipped with resistive heating elements, providing high thermal uniformity and a maximum operating temperature of 1280 °C. Heating is supplied by an external electric system controlled by a C 250 programmer, an electronic temperature controller with adjustable ramp rates. The SiO_2_ and NiO nanoparticles were incorporated into the pyrogallol-formaldehyde matrix at 20% and 1% mass ratios, respectively; the resulting wet gel was dried in open air at 50 °C for two weeks, then oven dried with a temperature ramp of 10 °C per day up to 150 °C to form to produce the THyNC. The synthesized materials NPOM, BHyNC and THyNC were thermally treated at 650 °C for two hours in a Carbolite programmed furnace tubular under an inert atmosphere. The temperature was increased at a controlled ramp rate of 5 °C min^−1^, followed by natural cooling to room temperature.

### 2.2. Characterization

The synthesized materials were subjected to comprehensive physicochemical characterization employing a suite of advanced analytical techniques. Crystallographic properties were analyzed *via* X-ray diffraction (XRD) using a MiniFlex benchtop diffractometer equipped with a graphite monochromator and Co-Kα radiation. The measurements were carried out at 40 kV and 30 mA, with a step size of 0.02° (2*θ*). Phase identification was performed by comparing the diffraction peaks with standard patterns from the JCPDS/ICDD database. Morphological and microstructural features were examined using Field Emission Gun-Scanning Electron Microscopy (FEG-SEM, JSM-7600F, SEI Resolution:1 nm at 15 kV 1.5 nm at 1 kV, in GB mode, 0.1 to 30 kV, probe current range : 1 pA to ≥200 nA and transmission electron microscopy (TEM, FEI Tecnai G2 F30, point: 2.0 angstrom line: 1.0 angstrom, 300 kV). X-ray Photoelectron Spectroscopy (XPS, Kratos Analytical, UK; SHIMADZU group) model: AXIS Supra (Two chamber ultra high vacuum system: analysis chamber (<2 10^−9^ Torr) and sample load-lock chamber (<5 10^−8^ Torr) equipped with an Al-Kα radiation source was employed to examine the surface elemental composition and chemical states of the samples. FTIR spectra were recorded using a JR7000 spectrometer (JEOL Inc.) in the range of 4000–400 cm^−1^ with a resolution of 1 cm^−1^. The samples were prepared as KBr pellets to ensure optimal signal quality. Raman spectroscopic analysis was performed using a Horiba Jobin-Yvon T64000 spectrometer with an green laser excitation wavelength of 532 nm. The measurements were carried out at room temperature, with a spectral resolution of 1 cm^−1^. The specific surface area, total pore volume and pore size distribution were characterized by nitrogen adsorption–desorption analysis, performed with a Micromeritics ASAP 2000 instrument. Thermogravimetric analysis (TGA) was performed using a Setaram Labsys™ system to evaluate the effect of thermal annealing on the weight loss of the sample prepared in the first step. Measurements were carried out under a nitrogen atmosphere with a heating rate of 5 °C min^−1^ up to 1000 °C. Electrical conductance was evaluated over the frequency range of 40 Hz to 1 MHz using an Agilent 4294 precision impedance analyzer with excitation voltage amplitude of 50 mV. In this electrical measurement, the samples were pressed into pellets with a thickness of 3 mm. A thin layer of silver was deposited on both parallel faces of each pellet to ensure ohmic contacts. Electrochemical measurements were performed in both the absence and presence of glucose to investigate the redox behavior and electrocatalytic activity of the materials. All measurements were conducted at a scan rate of 50 mV s^−1^ using a DropSens μStat 400 potentiostat/galvanostat at 25 °C in 0.1 MKOH solution.

## 3. Results and discussions

**Fig. 1:**
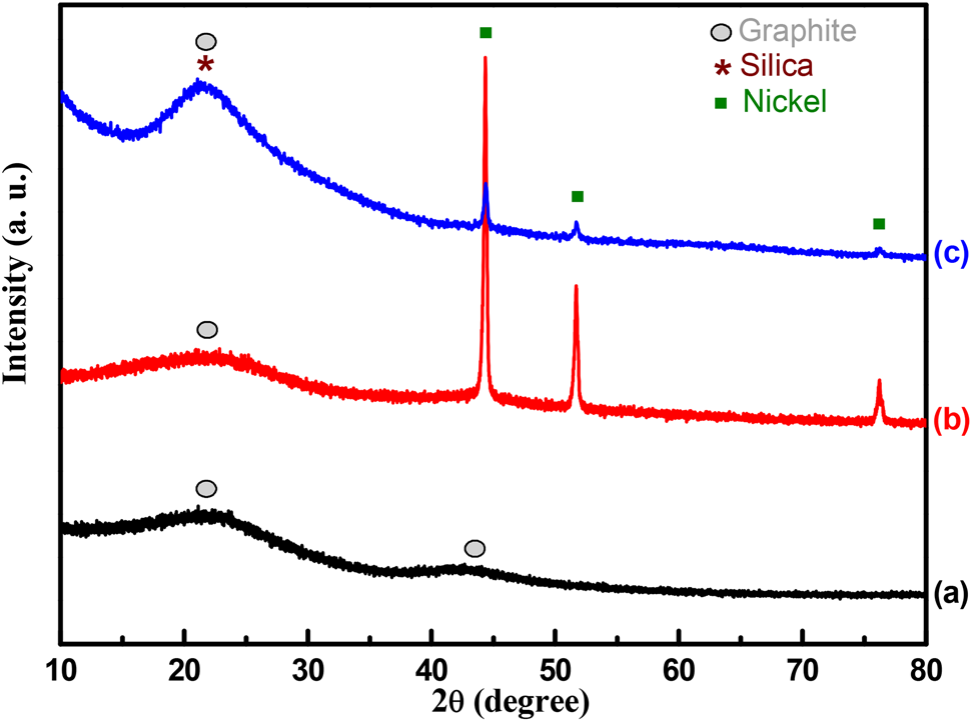
XRD diffractograms of; (a): NPOM, (b): BHyNC and (c): THyNC.

Fig. 1 presents the X-ray diffraction patterns of the different materials. The NPOM sample exhibits two broad diffraction features centered at approximately 22° and 44°, which are indicative of an amorphous carbon structure commonly associated with disordered graphite. In contrast, the BHyNC and THyNC samples retain the broad feature near 22°, while the one at 44° disappears, coinciding with the emergence of a sharp peak at around 44° corresponding to the (111) reflection of crystalline metallic nickel. Additionally, both BHyNC and THyNC display distinct diffraction peaks at approximately 51° and 76°, which can be attributed to the (200) and (220) planes of face centered cubic nickel, respectively. In the case of THyNC, the broad feature at 22° is likely a diffraction convolution from both amorphous silica and residual amorphous carbon suggesting the coexistence of these two disordered phases in the structure of THyNC.^45,46^

**Fig. 2:**
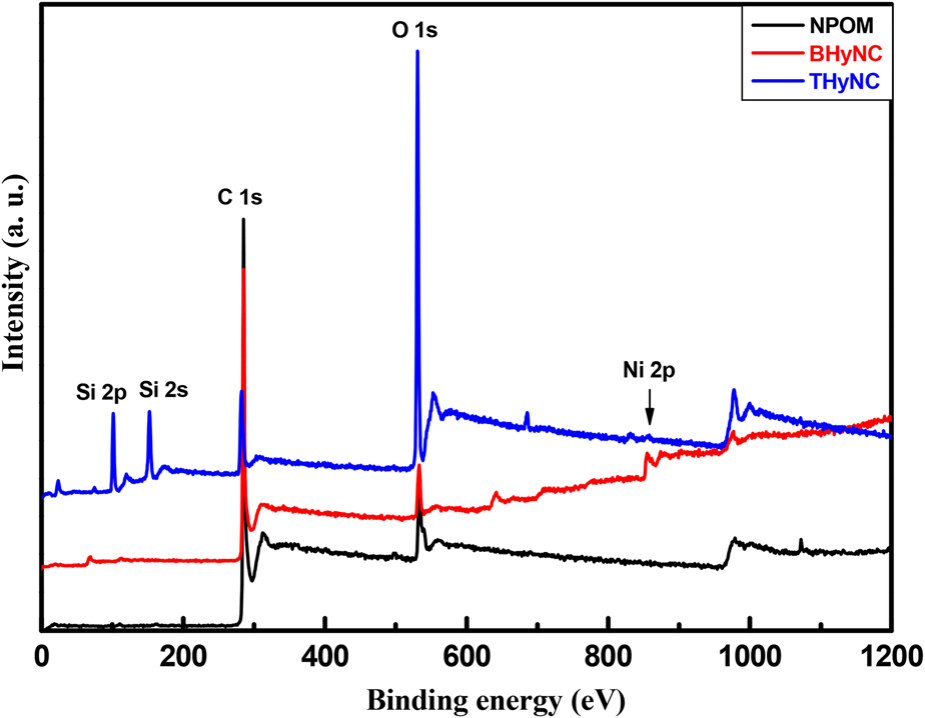
XPS spectra of different samples.

Fig. 2 depicts the XPS spectra of the three samples, revealing significant differences in surface elemental composition. The spectrum of NPOM predominantly shows a C 1s peak centered around 282.5 eV characteristic of graphitic carbon, along with a weak O 1s peak indicating the detection of oxygen containing functional groups. For BHyNC, a Ni 2p peak at 858 eV confirms the presence of metallic nickel species. In the case of THyNC, in addition to the C 1s, Ni 2p and O 1s peaks, two distinct silicon peaks Si 2p at 101.5 eV and Si 2 s at 152.2 eV are observed confirming the existence of silica nanoparticles.

The atomic ratios of O, Si, C and Ni in the different samples are presented in Table 1. For the NPOM sample, the atomic concentrations of carbon and oxygen are 87.05% and 12.94%, respectively. In the case of BHyNC, the concentrations of carbon, oxygen and nickel are 88.12%, 10.14% and 1.73%, respectively. For THyNC, the atomic concentrations are 33.15% for carbon, 46.19% for oxygen, 0.30% for nickel and 20.36% for silicon.

**Table 1:** Surface elemental concentration of various samples

Sample	C (at%)	O (at%)	Ni (at%)	Si (at%)
NPOM	87.05	12.94	—	—
BHyNC	88.12	10.14	1.73	—
THyNC	33.15	46.19	0.30	20.36

**Fig. 3:**
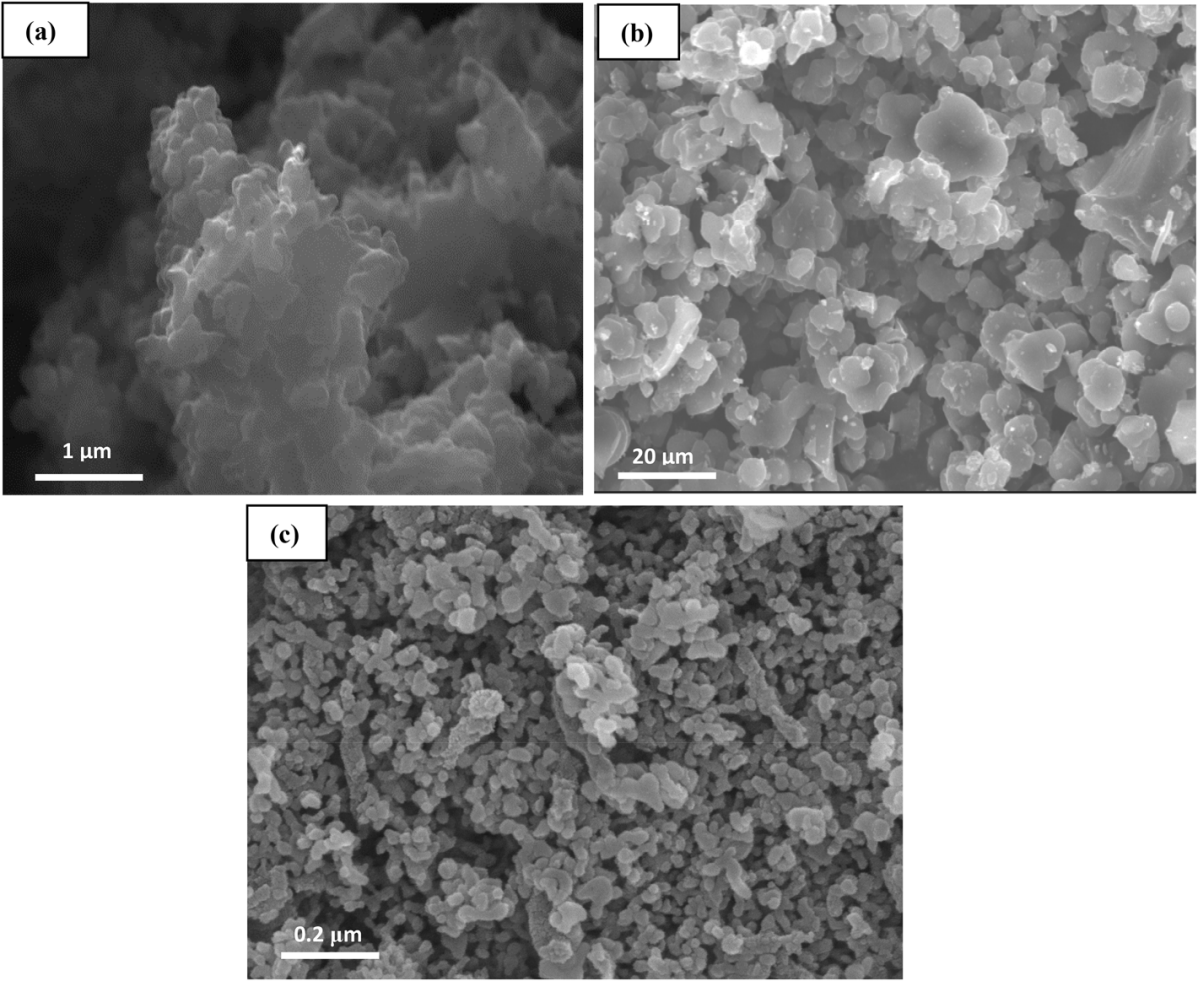
SEM images of; (a): NPOM, (b): BHyNC and (c): THyNC.

Fig. 3 shows the SEM images of the synthesized materials. Image (a), corresponding to the NPOM, reveals a homogeneous porous structure with uniformly distributed pores, which may indicate a high specific surface area. In the case of the BHyNC (image (b)), the incorporation of NiO has clearly altered the morphology of the matrix. Bright spherical microparticles are observed, suggesting the presence of nickel nanoparticles. Image (c) of the THyNC, shows a more complex structure with a fine distribution of particles, which could be attributed to the nanoscale dispersion of both nickel and silica phases.

The elemental composition of the materials was determined by Energy Dispersive X-ray spectroscopy (EDX) coupled with a scanning electron microscope. The mass fractions of carbon, oxygen, silicon and nickel in the various samples are summarized in Table 2. For the NPOM, C and O accounted for 98.28% and 1.72%, respectively. In the BHyNC, the measured mass fractions of C, O, and Ni were 77.67%, 14.7%, and 7.63%, respectively. For the THyNC, the mass fractions of C, O, Si and Ni were 26.82%, 46.72%, 25.36%, and 1.1%, respectively.

**Table 2:** Surface elemental concentration of different samples

Sample	C (wt%)	O (wt%)	Ni (wt%)	Si (wt%)
NPOM	98.28	1.72	—	—
BHyNC	77.67	14.7	7.63	—
THyNC	26.82	46.72	1.1	25.36

**Fig. 4:**
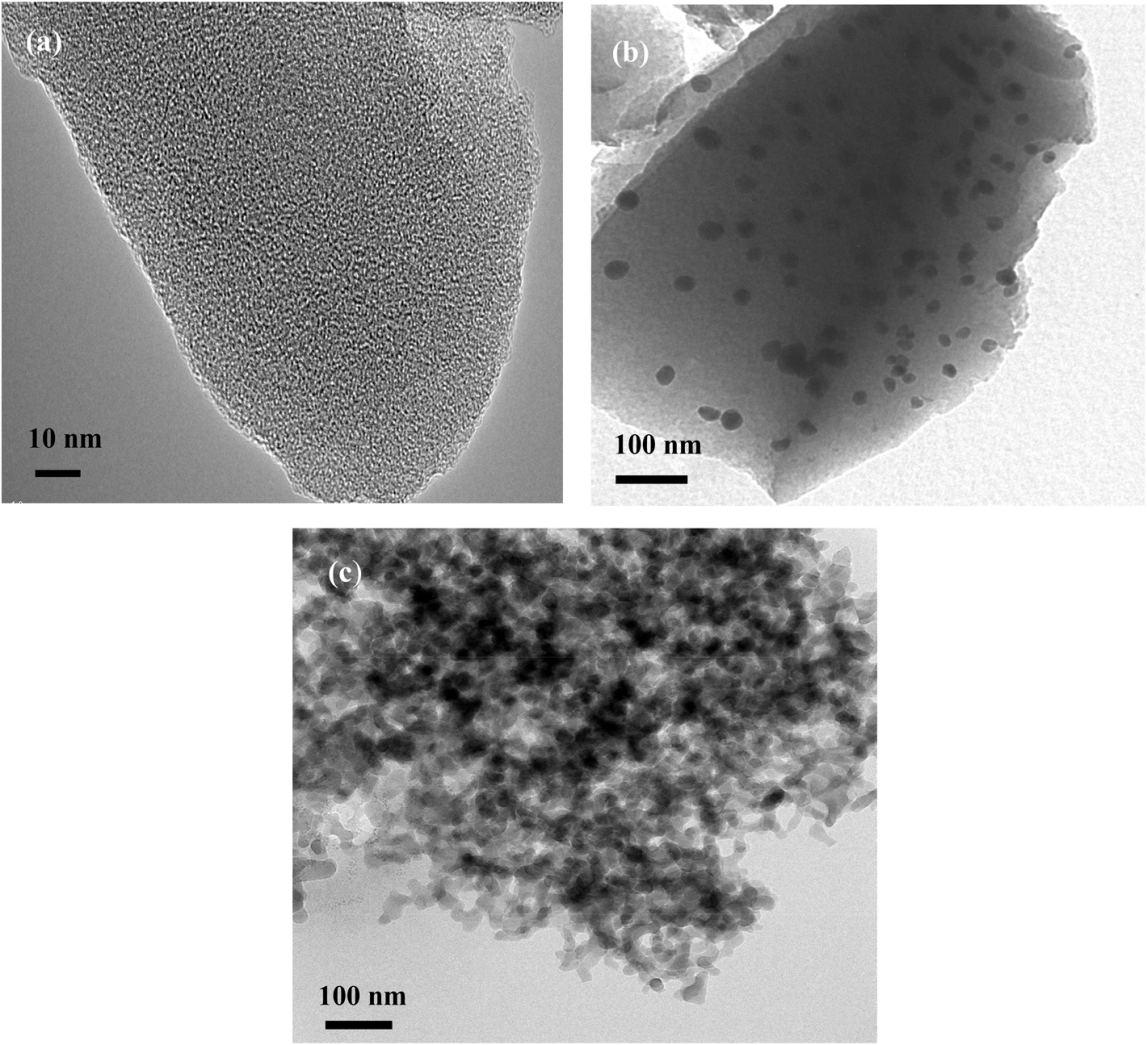
TEM images of; (a): NPOM, (b): BHyNC and (c): THyNC.

Fig. 4 displays the TEM images of NPOM (image (a)), BHyNC (image (b)) and THyNC (image (c)). Image (a), shows a homogeneous and nanoporous structure typical of NPOM. The diffuse contrast observed is indicative of an amorphous phase with uniformly distributed pores. In image (b), BHyNC exhibits well dispersed dark nanoparticles, attributed to nickel, embedded within the amorphous carbon matrix. Image (c), reveals a more complex nanostructure that corresponds to an agglomeration of two types of nanoparticles in the THyNC, where the dark regions correspond to metallic nickel and the lighter areas are likely associated with carbon and silica.

**Fig. 5:**
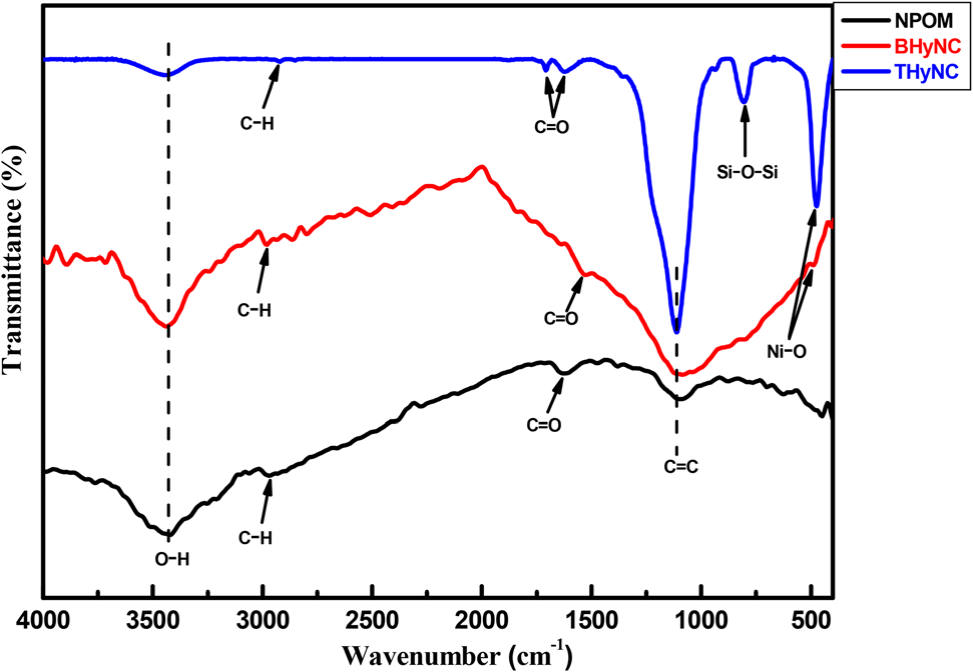
FTIR spectra of different samples.

The FTIR spectra shown in Fig. 5 highlight the structural and chemical evolution of our materials. The NPOM displays characteristic bands corresponding to O–H (around 3400 cm^−1^), C–H (approximately 3000 cm^−1^), C=O (around 1700 cm^−1^) and C=C (around 1100 cm^−1^) vibrations.^47–49^ Upon incorporating NiO nanoparticles into the carbon matrix (BHyNC), a new band appears around 570 cm^−1^, attributed to Ni–O vibrations, while the decreased intensity of the C=O and O–H bands suggests interactions between nickel and oxygen containing functional groups.^50,51^ In the case of THyNC, the presence of silica is confirmed by a strong band around 800 cm^−1^, corresponding to the Si–O–Si stretching vibration.^52,53^ The concurrent presence of Ni–O and Si–O–Si bands supports the formation of a ternary hybrid structure composed of carbon, nickel and silica.

**Fig. 6:**
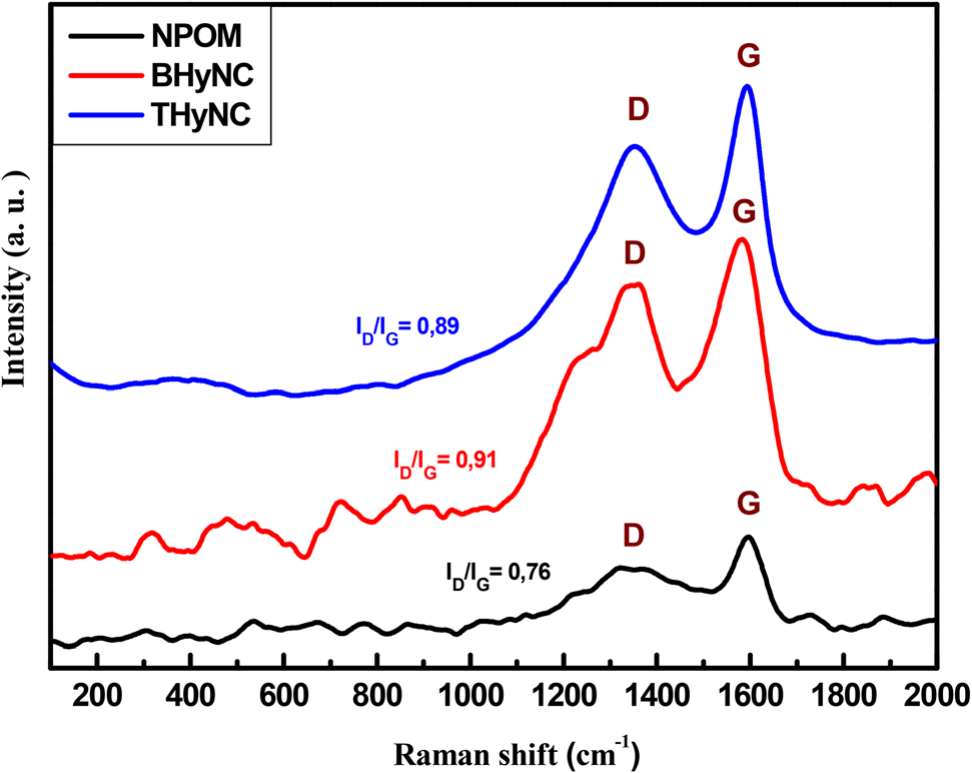
Raman spectra of different samples.

The Raman spectra of the different samples (Fig. 6) display two characteristic bands of carbon based materials: the D band at approximately 1350 cm^−1^, associated with structural defects and disorder in the sp^3^ hybridized carbon network and the G band around 1580 cm^−1^, corresponding to the vibrational modes of sp^2^ carbon atoms in well ordered graphitic domains.^54,55^ The *I*_D_/*I*_G_ intensity ratio serves as an indicator of the degree of disorder within the materials. This ratio is 0.76 for NPOM, 0.91 for BHyNC and 0.89 for THyNC. Based on the obtained *I*_D_/*I*_G_ values (0.7 <*I*_D_/*I*_G_ <1), it can be concluded that the various samples exhibit a disordered graphitic structure with the presence of structural defects. These values are considered acceptable when compared to those commonly reported in the literature, where *I*_D_/*I*_G_ ratios exceeding 1 typically indicate a structure predominantly composed of disordered carbon with a high defect density.^56–58^ Among the studied materials, NPOM exhibits the highest degree of graphitization, as evidenced by its lowest *I*_D_/*I*_G_ ratio. In contrast, the incorporation of NiO nanoparticles disrupts the graphitic framework, leading to an increased defect density in BHyNC. Notably, the addition of SiO_2_ nanoparticles in THyNC appears to mitigate these defects, suggesting that silica may have played a crucial role in promoting a more ordered carbon structure during synthesis or thermal treatment under an inert atmosphere.

**Fig. 7:**
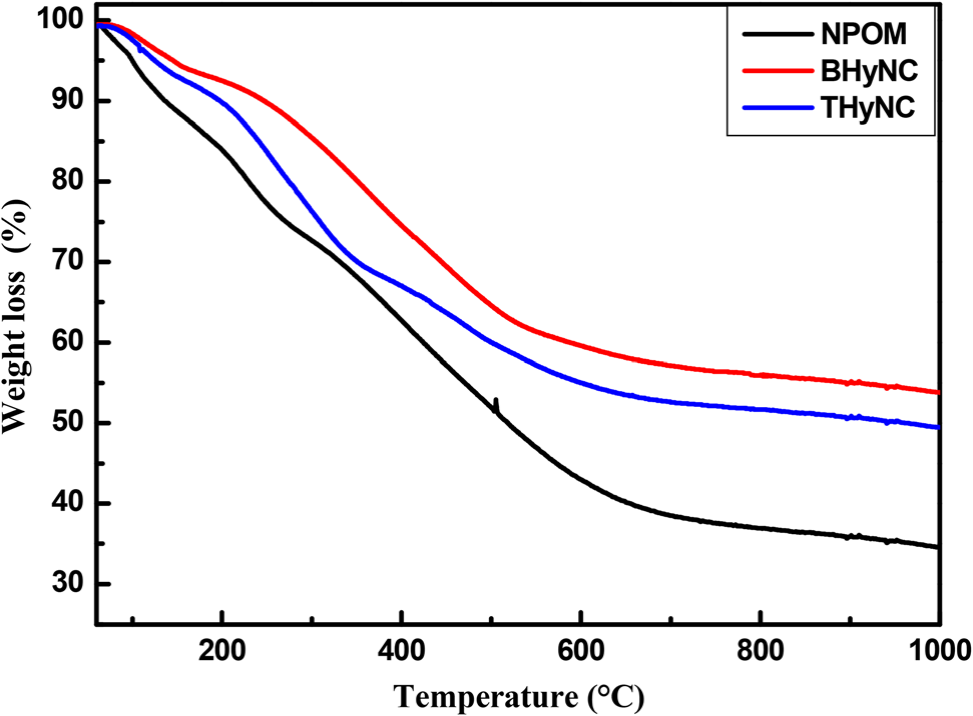
Thermogravimetric analysis (TGA) curves of different samples.

Fig. 7 presents the thermogravimetric analysis (TGA) of NPOM, BHyNC and THyNC, showing total mass losses of approximately 67%, 45% and 50%, respectively. Three distinct mass loss stages are observed: the first, between 40 and 130 °C, corresponds to the water desorption; the second, between 130 and 300 °C, is attributed to the desorption of residual precursors; and the third, above 300 °C, is related to decomposition and carbonization processes, leading to the formation of C–C bonds. The higher mass loss observed in NPOM indicates its lower thermal stability. In contrast, BHyNC shows reduced mass loss and enhanced thermal stability, which is attributed to the catalytic effect of nickel. In fact, at temperatures above 800 °C, Ni nanoparticles facilitate carbon structural reorganization and the formation of multi-walled carbon nanotubes, thereby reinforcing its stability.^59^ For THyNC, the presence of SiO_2_ promotes a uniform dispersion of Ni nanoparticles, thereby enhancing its electrocatalytic activity toward glucose oxidation, while resulting in slightly lower thermal stability compared to BHyNC.

**Fig. 8:**
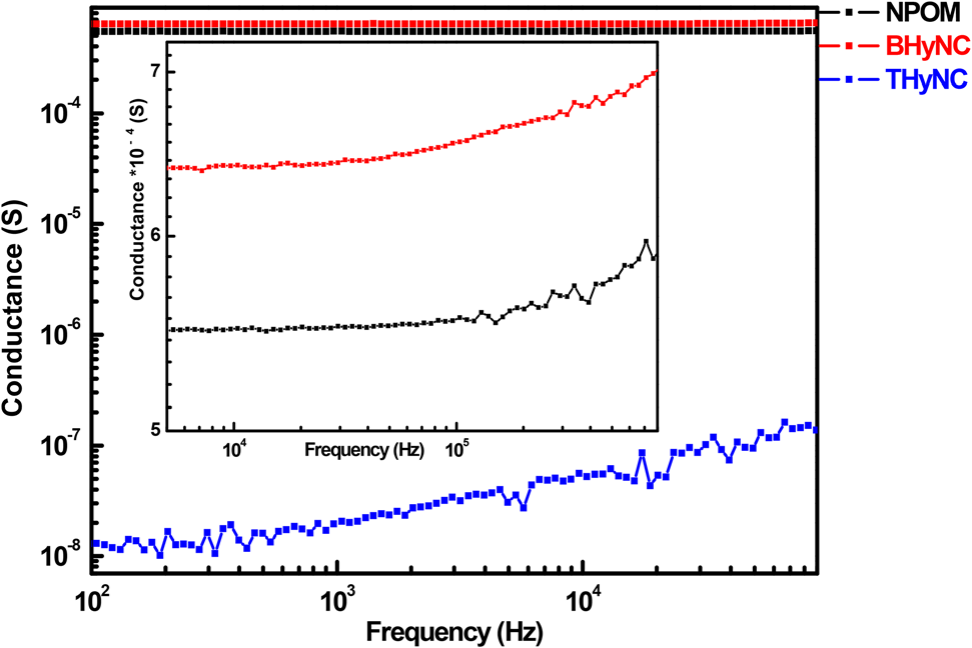
ac conductance at room measurement temperature of different samples.

The frequency dependent conductance of the NPOM, BHyNC and THyNC samples at room temperature follows Jonscher's universal model (Fig. 8). According to this model, the ac conductance comprises a dc component (*G*_dc_) and a frequency dependent term (*Aω*^s^).^60^ At low frequencies, a plateau corresponding to the dc conductance is observed, whereas at higher frequencies, the conductance increases according to a power-law, indicating a transport mechanism governed by localized hopping between trapped states. Among the three samples, BHyNC displays the highest conductance (around 5 × 10^−4^ S) across the entire frequency range, which is attributed to the presence of nickel nanoparticles that facilitate electron transport. Conversely, THyNC exhibits the lowest conductance (approximately 2 × 10^−8^ S), due to the insulating effect of silica nanoparticles that disrupt conductive pathways. However, the overall analysis suggests that nickel nanoparticles do not drastically improve conductivity in BHyNC compared to NPOM, as charge transport in these disordered carbon based materials primarily occurs between graphite nanoparticles.

**Fig. 9:**
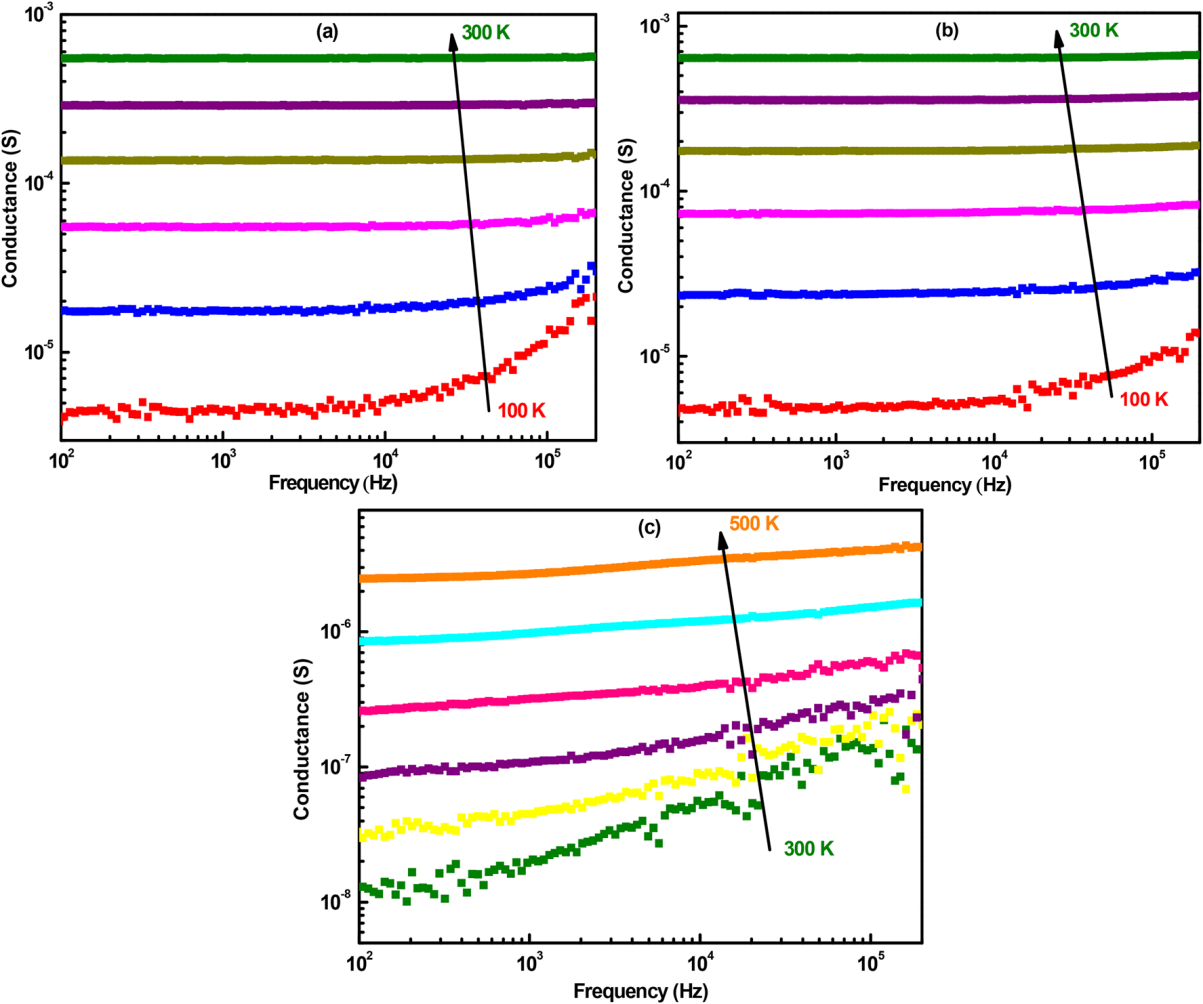
ac conductance at different measurement temperatures with a step of 40 K for; (a): NPOM, (b): BHyNC and (c): THyNC.

Based on this ac conductance study at room temperature and taking into account the detection limit of conductance for the impedance spectroscopy used (beyond 10^−9^ S), it can be concluded that NPOM and BHyNC can have a detectable response at low measurement temperatures. In contrast, THyNC can only become active for temperatures above 300 K. Therefore, ac conductance measurements were conducted over the range of 100 to 300 K for NPOM and BHyNC, and from 300 to 500 K for THyNC (Fig. 9). For all three materials, the conductance increases with both measurement temperature and frequency, reflecting their semiconductor behavior. The temperature dependent rise in conductance indicates a thermally activated transport mechanism, characteristic of hopping conduction between localized states. Notably, at low temperature (100 K), NPOM and BHyNC exhibit conductance values on the order of 10^−6^ S, comparable to that of THyNC measured at high temperature (500 K). This observation suggests that NPOM and BHyNC are well suited for low temperature applications due to their relatively high conductance at 100 K. Conversely, THyNC appears to be more appropriate for high temperature applications.

In general, for carbon based materials, porosity particularly pore volume has a direct influence on the variation of electrical conductivity. An increase in pore volume tends to disrupt the continuity of the electronic conduction network, resulting in a decrease in electrical conductivity.^61,62^ Nitrogen adsorption–desorption isotherms for the studied samples were obtained in previous work.^63,64^Table 3 summarizes the specific surface area, pore volume and pore size for NPOM, BHyNC and THyNC. NPOM exhibits the highest specific surface area (720 m^2^ g^−1^) along with a moderate pore volume (0.33 cm^3^ g^−1^). The presence of nickel in BHyNC leads to a decrease in both specific surface area (461 m^2^ g^−1^) and pore volume (0.22 cm^3^ g^−1^), likely due to partial pore blockage, which contributes to improved electrical conductance. In contrast, the addition of silica in THyNC significantly reduces the specific surface area (163 m^2^ g^−1^) while markedly increasing the pore volume (1.55 cm^3^ g^−1^), indicating a more open porous structure potentially 720 less favorable for electrical conduction but beneficial for molecular storage. The pore size shows a slight increase from 1.9 nm in NPOM to 2.6 nm in BHyNC, followed by a decrease to 1.4 nm in THyNC. These values reflect the microporous character of the NPOM and THyNC materials.

**Table 3:** Textural properties of various samples

Sample	Specific surface area (m^2^ g^−1^)	Pore volume (cm^3^ g^−1^)	Pore size (nm)
NPOM	720	0.33	1.9
BHyNC	461	0.22	2.6
THyNC	163	1.55	1.4

**Fig. 10:**
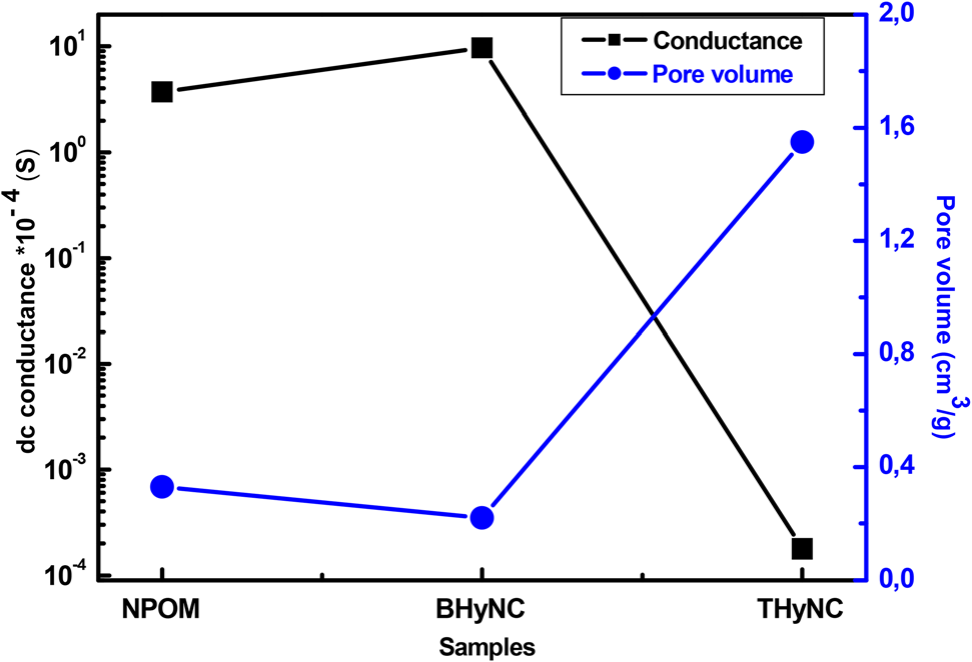
Inverse variation of dc conductance and pore volume.

Fig. 10 illustrates an inverse relationship between the dc electrical conductance (at 100 Hz) and the pore volume for the NPOM, BHyNC and THyNC materials. It is evident that as the pore volume decreases, the electrical conductivity increases.^65,66^ Specifically, NPOM exhibits the largest pore volume and the lowest conductance, whereas THyNC, having the smallest pore volume, shows the highest conductance. This inverse correlation can be attributed to the fact that a higher pore volume disrupts the continuity of the electrical network, impeding charge transport, while a denser, less porous structure facilitates improved electrical conduction.

**Fig. 11:**
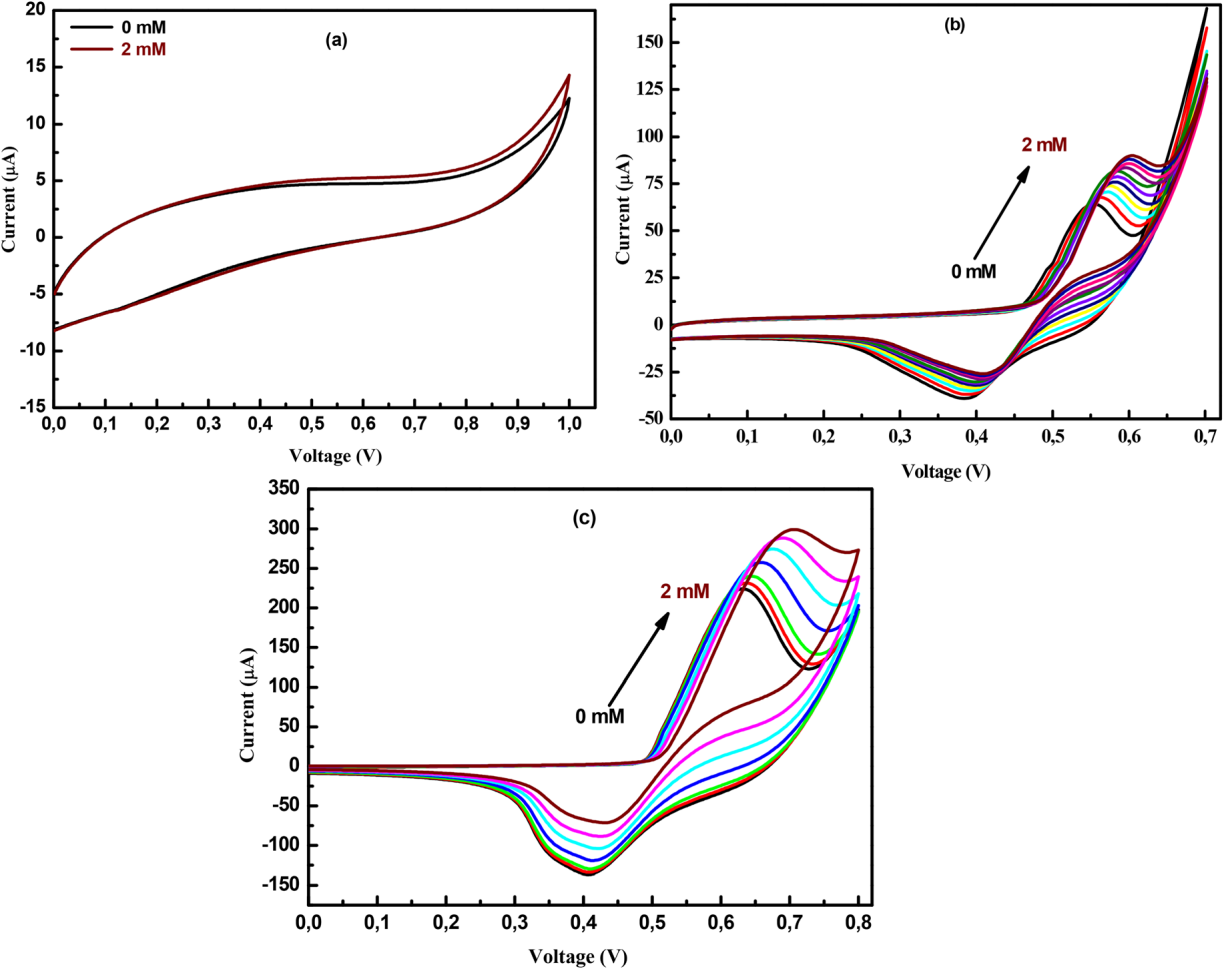
Current–Voltage characteristics for different glucose concentrations of; (a): NPOM, (b): BHyNC and (c): THyNC.

The electrochemical behavior of NPOM, BHyNC and THyNC towards glucose oxidation was investigated in a 0.1 M KOH solution using current–voltage measurements. Fig. 11a shows the current–voltage curves recorded in both the absence and presence of 2 mM glucose for NPOM. No anodic or cathodic peaks were observed under either condition, indicating that NPOM exhibits negligible electrocatalytic activity towards glucose oxidation. In contrast, for BHyNC and THyNC (Fig. 11b and c), distinct oxidation and reduction peaks were observed in both the absence and presence of glucose at concentrations ranging from 0 to 2 mM. These peaks can be attributed to the electrochemical redox reaction of the Ni^2+^/Ni^3+^ couple at the electrode surface.^67^ The anodic peak current increases and shifts slightly towards more positive potentials as the glucose concentration increases. This behavior suggests an extended oxidation process at the regenerated NiOOH sites following the initial glucose oxidation at the electrode surface. Conversely, the cathodic peak current tends to decrease as the glucose concentration increases, which is probably related to the consumption of NiOOH species during the glucose oxidation process.^68^

**Fig. 12:**
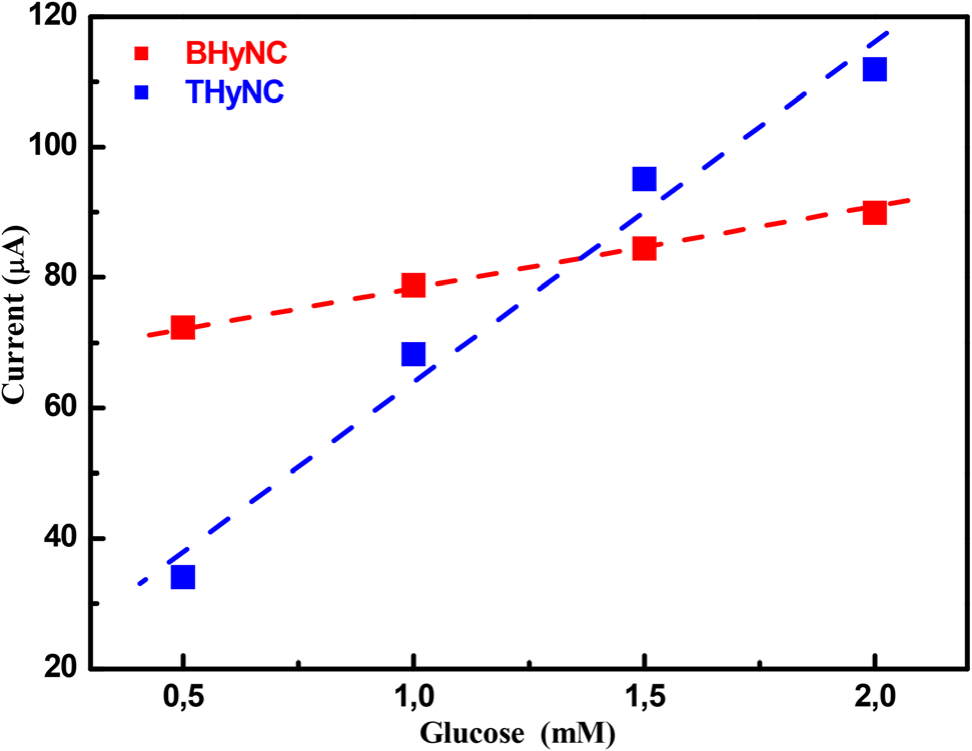
Calibration curves of BHyNC and THyNC at different glucose concentrations.

Fig. 12 presents the variation of oxidation current as a function of glucose concentration of the anodic peaks observed at 0.55 V and 0.6 V for the BHyNC and THyNC, respectively. A linear increase in oxidation current is observed with increasing glucose concentration, as indicated by the calibration curves. The linear regression for glucose concentrations ranging from 0.5 to 2 mM yielded sensitivities of approximately 93 μA mM^−1^ cm^2^ for BHyNC and 416 μA mM^−1^ cm^2^ for THyNC. The higher sensitivity of THyNC compared to BHyNC, despite the electrochemical inactivity of silica, can be attributed to the role of SiO_2_ nanoparticles as a structural support. By filling the pores of the NPOM, SiO_2_ promotes a more homogeneous dispersion of Ni nanoparticles and prevents their agglomeration. This effect arises from both chemical and physical contributions: surface hydroxyl groups of SiO_2_ act as anchoring sites that immobilize Ni nanoparticles and guide their nucleation, while its porous structure and high surface area act as a physical barrier that limits particle growth during pyrolysis. As a result, Ni nanoparticles remain uniformly distributed within the matrix, ensuring a higher density of electroactive sites and enhanced glucose sensing performance.

Aiming to evaluate the performance of the synthesized samples, Table 4 provides a comparison with recently reported materials used for non-enzymatic glucose sensing. Advanced systems, such as silver-doped quaternary ZrO_2_/mesoporous silica-graphene nanocomposites^69^ and mesoporous silica-coated graphene nanosheets,^70^ have demonstrated exceptionally high sensitivities (9000 and 970 μA mM^−1^ cm^2^, respectively) along with extremely low detection limits (0.05 and 0.03 μM, respectively). However, these systems typically exhibit relatively narrow linear ranges (0.05–0.35 mM and up to 0.9 mM), which limits their practical applicability for glucose monitoring. Similarly, Cu/g-SiCNT/CuO composites^71^ showed remarkable sensitivity (2051 μA mM^−1^ cm^2^), yet with a constrained linear range of 1 μM to 4 mM. In contrast, 3D ternary Cu_2_O/MXene/rGO composites^72^ offered a more balanced performance, combining moderate sensitivity (264 μA mM^−1^ cm^2^) with a wide linear range (0.1–14 mM), underscoring the advantage of 3D architectures in enhancing charge transport and species diffusion. Other recent works, such as Cu-decorated laser-induced graphene,^73^ presented moderate sensitivity (223 μA mM^−1^ cm^2^) with a limited linear range of 0.1–1 mM, while PEDOT/MXene/GQD nanocomposites^74^ achieved extremely high sensitivity (5410 μA mM^−1^ cm^2^) but only within a very narrow linear range (up to 0.5 mM) and with a relatively high detection limit (44.6 μM). Our BHyNC and THyNC materials exhibit sensitivities of 93 and 416 μA mM^−1^ cm^2^, respectively, within the 0.5–2 mM range. Although the linear range is slightly narrower, the sensitivity of THyNC exceeds that of previously reported carbon based nanocomposites^75,76^ and it is comparable to-or even higher than-some recent MXene and LDH based sensors.^77^ These results indicate that the presence of SiO_2_ effectively disperses Ni nanoparticles and increases the density of electroactive sites, resulting in significant electrocatalytic activity. Overall, while ultra-high sensitivity can be achieved in some systems, THyNC provides a promising balance of sensitivity, stability and a practical linear detection range, while maintaining the advantage of a relatively simple and cost-effective synthesis approach.

**Table 4:** Comparative glucose sensing performance of BHyNC and THyNC with recently reported materials (2021–2025)

Material	Sensitivity (μA mM^−1^ cm^2^)	Linear range	Limit detection (μM)	Reference
Silver doped ZrO_2_ coupled graphene based mesoporous silica quaternary nanocomposite	9000	0.05 to 0.35 mM	0.05	69
Mesoporous silica coated graphene oxide nanosheet	970	0 to 0.9 mM	0.03	70
Cu/g-SiCNT/CuO nanocomposite	2051	1 μM to 4 mM	0.8	71
3D ternary composite Cu_2_O/MXene/rGO	264	0.1 to 14 mM	1.1	72
Cu-decorated laser induced graphene from natural cork	223	0.1 to 1 mM	9.7	73
PEDOT/MXene/GQD nanocomposite	5410	0 to 0.5 mM	44.6	74
NiO/C@rGO nanocomposite derived from Ni (gallate)	23	1 μM to 1 mM	0.6	75
SWCNTs-mesoporous silicon nanocomposite	0.06	0.5 to 28 mM	9.6	76
MXene and nickel cobalt layered double hydroxide (NiCo-LDH)	154	1 μM to 4 mM	0.2	77
BHyNC	93	0.5 to 2 mM	10	This work
THyNC	416	0.5 to 2 mM	30	This work

**Fig. 13:**
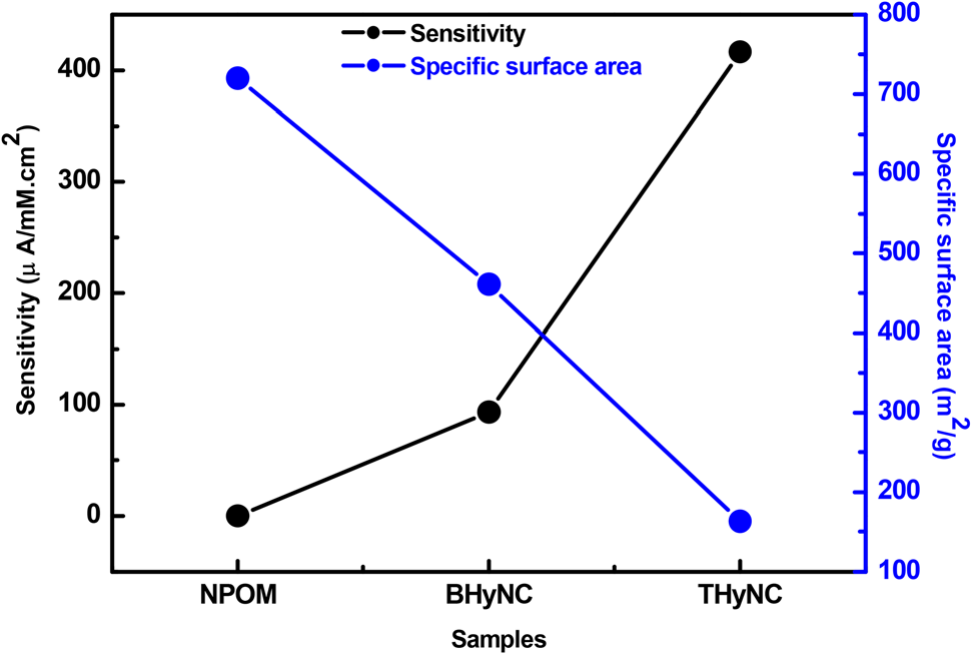
Inverse variation of sensitivity and specific surface area.

Fig. 13 shows an inverse relationship between sensitivity and specific surface area across the different samples. NPOM displays the largest specific surface area but negligible sensitivity, indicating low electrochemical activity despite its highly developed surface. In contrast, BHyNC presents a moderate specific surface area with a markedly higher sensitivity, highlighting the electrocatalytic activity of nickel toward glucose oxidation. Notably, THyNC, which has the smallest specific surface area, demonstrates the highest sensitivity.

These results indicate that sensitivity is primarily determined by the effective dispersion of catalytically active Ni nanoparticles rather than by the specific surface area. Generally, materials with a higher specific surface area provide more active sites and thus greater selectivity, as reported in the literature.^78,79^ In our study, however, performance is mainly governed by the nature and accessibility of the catalytic sites rather than by surface area alone. For instance, in NPOM, despite its high surface area, the limited electrochemical activity of carbon restricts sensitivity. Upon incorporation of NiO nanoparticles, as in BHyNC, the surface area decreases slightly, but the presence of catalytically active Ni sites markedly improves sensitivity. In ThyNC, the surface area is further reduced due to its denser structure; nevertheless, the uniform dispersion of Ni nanoparticles, facilitated by SiO_2_, ensures superior electrochemical efficiency and thus the highest sensitivity. In summary, it is the quality and distribution of active sites, rather than the specific surface area, that predominantly govern sensor performance in these pyrogallol-formaldehyde based materials.

## 4. Conclusion

This study demonstrated that the incorporation of NiO and SiO_2_ nanoparticles significantly modifies the structural, morphological, vibrationnel, electrical and electrochemical properties of a nanoporous organic matrix (NPOM). While the addition of NiO improves electrical conductance and introduces electrocatalytic activity, the co-incorporation of SiO_2_ further enhances the dispersion of nickel nanoparticles, leading to superior glucose sensing performance. The ternary hybrid nanocomposite (THyNC) exhibited the highest sensitivity (416 μA mM^−1^ cm^2^) despite having the lowest specific surface area, highlighting that sensitivity is more influenced by the effective dispersion of catalytically active nanoparticles. In contrast, the electrical conductance decreased with increasing pore volume, with THyNC showing the lowest conductance due to the insulating effect of SiO_2_ nanoparticles. On the whole, THyNC emerged as the most promising material for non-enzymatic glucose sensor applications, while NPOM and BHyNC demonstrated potential for low-temperature electronic applications. The study successfully established the critical relationship between textural properties, electrical behavior and electrochemical performance in these based pyrogallol-formaldehyde materials.

## Conflicts of interest

The authors declare that they have no conflict of interest.

## Author contributions

N. Ben Mansour performed the synthesis of the materials, contributed to the interpretation of the results, and wrote the manuscript. M. Hjiri, N. Mustapha and Fatemah M. Barakat were involved in material characterization and data analysis. Talal F. Qahtan and L. El Mir contributed to the interpretation of the results and manuscript revision.

## Data Availability

All data are available on request.
